# Patient Perceptions of Decision-making and Quality-of-life Following Surgical Resection of Pancreatic Adenocarcinoma: A Mixed-methods Study

**DOI:** 10.1097/AS9.0000000000000214

**Published:** 2022-10-25

**Authors:** Josh Bleicher, Aubrey Place, Alex H. S. Harris, Courtney L. Scaife, Lyen C. Huang

**Affiliations:** * Department of General Surgery, University of Utah, Salt Lake City, UT; † Department of Surgery, Stanford University, Stanford, CA; ‡ Center for Innovation to Implementation, VA Palo Alto Healthcare System, Palo Alto, CA; § Huntsman Cancer Institute at the University of Utah, Salt Lake City, UT, USA

**Keywords:** mixed-methods research, quality-of-life, qualitative research, pancreatic adenocarcinoma, surgical oncology

## Abstract

Pancreatic adenocarcinoma (PDAC) remains a highly lethal disease, with surgery, the only opportunity for cure, accompanied by high rates of morbidity. Understanding patients’ lived experiences after surgical resection of PDAC is critical to knowing whether the decision to undergo surgery was worth it for these patients.

**Methods:**

We performed a convergent, mixed-methods study with patients who underwent resection of PDAC between January 1, 2019, and January 8, 2020. Quantitative data (medical record review and 3 questionnaires) were analyzed using descriptive statistics. Qualitative data (semistructured interviews) were analyzed using the constant comparative method. Data were then compared for congruence.

**Results:**

Eighteen of 22 eligible participants completed interviews and 11 completed questionnaires. Data collection occurred at a median of 14.2 months (IQR 11.6–16.3) from surgery. We identified 4 main themes. First, persistent negative symptoms were common for patients, but patients adapt to these and are satisfied with their “new normal.” Second, patients have varied and continually evolving mindsets throughout their cancer journey. Third, despite decreased quality-of-life, patients have a high degree of satisfaction with their decision to pursue surgery. Finally, patients were okay with a passive role in decision-making around surgery. Despite variable involvement in decision-making and outcomes, no participants reported regret over the decision to pursue surgery.

**Discussion:**

This nuanced account of patients’ lived experiences following surgery for PDAC allows for an improved understanding of the impact of pancreatic resection on patients. Surgeons can use these data to improve preoperative counseling for patients with PDAC and help guide them to making the correct decisions about surgery.

## Abstract 2

Mini-abstract: Patients experience persistent, life-limiting symptoms following pancreatic resection for pancreatic adenocarcinoma. Patients adapt to live with this new normal however, and are highly satisfied with their decisions to pursue surgery. Surgeons should emphasize these lifestyle changes during preoperative counseling.

## INTRODUCTION

Decision-making around high-risk medical therapies for highly lethal cancers is complex and physicians sometimes struggle to guide patients through this decision-making process. The risks and benefits of pancreatic resection for pancreatic adenocarcinoma (PDAC) represent one example of this dilemma. PDAC is one of the most highly lethal cancers, with 5-year overall survival around 9%, improved only slightly from 5% survival rates 20 years ago.^1^ Surgical resection of PDAC represents the only chance for cure or prolonged survival, with 5-year overall survival rising to 25% in patients with resectable disease.^2^ Advances in pancreatic surgery, including minimally invasive techniques and vascular reconstruction, have expanded the number of operative candidates and decreased operative morbidity and mortality^3–5^; however, the risks of pancreatic resection remain high, with major complications occurring in up to 30% of patients following surgery.^6,7^

Even without immediate postoperative complications, pancreatic resection for PDAC is a life-changing operation with lasting impact on patients’ quality-of-life (QOL). Prior studies have demonstrated the long-term impacts of pancreatic resection on patients’ daily lives, with persistent digestive abnormalities, including bloating, indigestion, flatulence, altered bowel habits, and decreased energy.^8,9^ Understanding the influence these symptoms have on patients’ daily lives is unclear however. Moreover, little is understood about how patients view the tradeoffs presented by the risks and benefits of pancreatic resection for PDAC.

Understanding patients’ lived experiences following pancreatic resection for PDAC can help physicians guide patients to make more informed decisions. To help future patients faced with this decision, we asked patients to describe their experiences with pancreatic surgery and to explain how they weighed the complex risks and benefits of surgery when deciding on a treatment plan. We asked patients to reflect on their decisions to undergo pancreatic resection and retrospectively determine, with full knowledge of their clinical and oncologic outcomes and impact on QOL, whether or not it was worth it.

## METHODS

We performed a convergent, mixed-methods study with concurrent quantitative and qualitative data collection. For qualitative data, participants completed a single, approximately 60-minute long interview. Quantitative data sources consisted of review of a prospectively collected institutional cancer registry and the electronic medical record (EMR), and 3 different participant-completed questionnaires.

### Participants

All English-speaking, adult patients (age >18) who underwent pancreatic resection for a diagnosis of PDAC between January 1, 2019, and January 8, 2020, were included. Data from the institutional cancer registry and EMR was obtained for all patients. Eligible participants were then approached for the qualitative and questionnaire portions of the study between 6 and 18 months postoperatively. When possible, patients were contacted about study participation. If patients were deceased, their next-of-kin was contacted for participation. Patients were excluded from qualitative and questionnaire data collection if they could not be reached based on available contact information or if they did not speak English. All participants were mailed information about the study, then called on 2 separate occasions for possible recruitment. Informed consent was obtained from all participants. This study was approved by the University of Utah Institutional Review Board, identification number 00131579.

### Data Collection

A general surgery resident with training in qualitative interviewing (JB) conducted semistructured interviews, approximately 1 hour in length, with all participants. Interviews were conducted virtually via telephone or video, with all audio recorded. An interview guide was created to gauge patient perceptions on their recovery from surgery, their quality-of-life, their mindset toward their cancer diagnoses and their decisions to pursue treatment, and the medical care they received (Supplement 1, http://links.lww.com/AOSO/A176). Questions on recovery from surgery were created from review of validated questionnaires used to assess wellbeing following pancreas surgery. Questions on patient mindset and decision-making were developed with input from experts in pancreatic surgery, palliative medicine, and qualitative methodology. All questions were open-ended. The interview guide was modified slightly for use with patient’s next-of-kin. Participants did not receive compensation for participation. All interviews were then transcribed verbatim.

Living patients also completed 3 questionnaires, the Decision-Regret Scale (DRS), the Controlled Preferences Scale (CPS), and the SDM-Q-9 (a 9-item shared decision–making questionnaire). The DRS is a validated, 5-question survey that helps identify the degree of regret patients experience over a specific healthcare decision—pancreatic resection in this study.^10^ This is scored on a scale from 0 to 100, with 100 indicating high regret and low scores indicating satisfaction with decision-making. The CPS uses a single question to allow participants to describe the role they like to have in their medical decision-making.^11^ There are 5 choices, ranging from a completely passive role (E), where doctors make all treatment decisions, to a completely active role (A), where patients make all treatment decisions. The middle choice (C) describes a collaborative role where decisions are made together. The SDM-Q-9 is a validated questionnaire that uses a 6-point Likert scale to assess patient satisfaction with their role in decision-making around a healthcare decision.^12^ Scores are reported as mean response per item (range 1–6), with higher scores indicating a greater degree of satisfaction.

Finally, information about patient’s clinical history was obtained through review of the EMR and a prospectively maintained, institutional database. This included information on surgical variables, such as type of procedure, presence of anastomotic leak, and length of stay, and oncologic variables, including recurrence and overall survival.

### Data Analyses

Interviews were analyzed using the constant comparative method, an inductive approach to qualitative analysis.^13,14^ Key themes and concepts from interview transcripts were recorded as codes during review of a random sample of 2 interviews by 2 investigators (JB, AP). This initial set of codes was then revised based on discussion with other members of the research team and analysis of further interviews. We continued to analyze transcripts in this fashion until all themes were represented by unique codes and a high degree of inter-rater reliability had been reached (Cohen’s kappa ≥0.7 for all codes).^15^ After this process was complete, the remaining interviews were coded independently by 1 of 2 investigators (JB, AP). Final codes were then reviewed by the research team to identify key themes from the interview transcripts. In accordance with qualitative methodology, quantification of codes was not performed, as not all participants are asked the same questions when using a semistructured interview guide.

Quantitative data were first analyzed using descriptive statistics. Clinical characteristics of patients who were interviewed and not interviewed were compared to minimize concern for selection bias. This was performed using the chi-square test or Fisher’s exact test, as appropriate. Two-sided *P* values <0.05 were considered statistically significant. Survey data are described without further analysis secondary to the low number of participants. Stata Version 15.1 (Stata Corp, College Station, TX, USA) was used for all statistical analyses. Qualitative and quantitative data were then compared to identify trends and evaluate the level of congruence between findings.

## RESULTS

During the study time period, 29 patients underwent pancreatic resection for PDAC. Patients were excluded if they did not have up-to-date contact information (n = 6) or did not speak English (n = 1). Interviews were completed with 18/22 (81.8%) participants eligible for inclusion. Most interviews were performed with patients (n = 16, 88.9%). Two patients had died prior to study eligibility (deaths at 1.2 and 5.0 months from resection), and their next-of-kin were interviewed about their experience. Most participants were male (n = 13, 72.2%) and median age at the time of resection was 71.6 (Interquartile Range [IQR], 66.1–75.3). Fourteen patients underwent PD and 4 distal pancreatectomy. Interviews took place at a median of 18.9 months (IQR, 15.0–20.7) from pancreatic cancer diagnosis and 14.2 months (IQR, 11.6–16.3) from pancreatic resection. Three patients developed recurrence prior to their interview date (at 2.9, 4.3, and 16.4 months from resection). Interviewed patients did not differ significantly in treatment or oncologic outcomes from all patients who underwent pancreatic resection for pancreatic adenocarcinoma at the time of study recruitment (Table 1).

**Table 1.** Demographic and Clinical Details of All Eligible Participants

**Variable**		**All(n = 29**)	**Interviewed(n = 18**)	**Not Interviewed(n = 11**)	***P***
Sex	Male	21 (72.4)	13 (72.2)	8 (72.7)	0.98
	Female	8 (27.9)	5 (27.8)	3 (27.3)	
Age at resection (years)	Median (IQR)	70.2 (61.9–74.5)	71.6 (66.1–75.3)	63.5 (59.0–72.3)	0.41
Stage at diagnosis	IA	4 (14.3)	2 (11.8)	2 (18.2)	0.91
	IB	10 (35.7)	7 (41.2)	3 (27.3)	
	IIA	1 (3.6)	1 (5.9)	0 (0)	
	IIB	5 (17.9)	3 (17.7)	2 (18.2)	
	III	9 (28.6)	4 (23.5)	4 (36.4)	
Neoadjuvant chemotherapy		23 (79.3)	14 (77.8)	9 (81.8)	0.59
Operation	PD	24 (82.8)	14 (77.8)	10 (90.9)	0.62
	DP	5 (17.2)	4 (22.2)	1 (9.1)	
Anastomotic Leak		3 (10.7)	2 (11.1)	1 (10.0)	1.00
Length of stay (days)	Median (IQR)	6 (6–8)	6.5 (5–8)	6 (6–10)	0.51
Adjuvant chemotherapy		10 (34.5)	6 (33.3)	4 (36.4)	0.66
Recurrence		5 (17.2)	3 (16.7)	2 (18.2)	1.00
Deceased		3 (10.3)	2 (11.1)	1 (9.1)	1.00

DP indicates distal pancreatectomy; IQR, interquartile range; PD, pancreaticoduodenectomy.

### Physical Recovery From Surgery

Some participants felt surgery had no negative effect on their lives and reported feeling remarkably well through the perioperative period. Participant 01 stated “it was sort of a piece of cake.” However, most participants described a period of difficulty following surgery. The most frequently reported complaints were fatigue, low energy, weakness, and digestive symptoms (Table 2). Digestive symptoms included bloating, loose stools, anorexia, and weight loss. Pain was rarely a major complaint, and most participants denied having significant issues related to pain beyond the first 1–2 weeks after surgery. Other physical complaints, including fevers, itching, jaundice, or problems with concentration were rare.

**Table 2.** Qualitative Examples Portraying Patient Perceptions of their Physical Symptoms Following Pancreatic Resection

**Symptom**	**Exemplary Quotes**
Physical Function	“I do stiff physical work, but I don’t last only an hour or two. I’m short of breath…I have no stamina whatsoever.”—Participant 03 “Since my surgery, I get tired a little faster and I have to stop and I will take a nap during the day and I just, 90 percent of the time I feel pretty good, but then there’s a few days that I’m just wiped out, I don’t have any energy and I just kind of lay around the house.”—Participant 09 “Oh, I just don’t do very much. I mess around a little bit and then I sit down and take five. I might get up and do something else and I might not.”—Participant 10
Appetite	“[Food] just don’t sound good to me, and I can make some good food or get some good food, but... It’s not money or, you know, it’s not—that’s not the problem, that we don’t have food. It’s just that I—it’s hard for me to eat it.”—Participant 05 “After the surgery. Before I weighed 265 pounds. And after the surgery I went down to around 170. And I didn’t have any appetite, and so they put a feeding tube down me, and I was on the feeding tube for, oh, six, seven, eight, nine months.”—Participant 13 “Well, he couldn’t eat, so he constantly, you know, he went from about 170, 180, to a buck 10. You know, skin and bones. You know, had no strength, you know, because he couldn’t eat. You know…his dietary thing was a big issue after the surgery, because he could never eat. If he did, he was always in pain. Even after the surgery.”—Participant 16
Diarrhea, Bloating, Gas	“Digestive problems with basically diarrhea and gas pains. I mean, that’s a daily thing. It goes on almost 24/7. So that’s the difference in my life.”—Participant 02 “I had nausea almost every day, and diarrhea went on for months, months. It was just like, here we go with this crap again.”—Participant 04 “I have a lot more loose stool, and lots more gas. I’m pretty much back to normal other than the gas from the surgery. And we’re still—you know, we’re working on that.”—Participant 17
Pain	“No, I think the pain is pretty much, what happened there is gone.”—Participant 04 “As far as the stitches and all that, it never hurt. Never did. Never bothered me. Must be one of them kind of people, I guess.”—Participant 06 “Well, while I was in the hospital I had bouts of [pain]…, it was not by any stretch like excruciating pain or anything like that. It didn’t cause me to have to catch my breath or anything. It was I think managed pretty well. The first few days just moving around of course I had pain from the incision. When I got home I had a couple of prescriptions. One was an opioid, and I think I used that as prescribed but for only about eight days… I didn’t really have a lot of pain. It was very manageable and, yeah, not too bad.”—Participant 07

This initial period of recovery varied between participants but was typically 6–8 weeks long. Most participants described a slow, but gradual recovery during this time period, with participants judging their experience of recovery primarily by their change in activity level and appetite. While most participants did not recover to a point where they felt their health was equivalent to their preoperative health, they did report getting to a point where their QOL was acceptable to them. Participants frequently described how they altered their lives to accommodate their new, persistent symptoms. This included avoiding certain foods, changing routines to avoid prolonged periods of activity, or limiting social interactions based around meals. Participants frequently described getting to a “new normal,” a way to function day-to-day and maintain an adequate QOL while accepting the alterations in their bodies and lifestyles. At times, these ongoing symptoms were life-limiting; however, participants described an acceptance of their new normal as an acceptable price to pay for the opportunity to prolong life.

Participants who experienced complications described a delay in getting to this new normal; however, at the time of interview, these participants did not report experiencing life in a substantially different way than those who did not experience complications. Next-of-kin interviewed on the experience of the 2 patients who died within 6 months of surgery described a period of prolonged suffering. These 2 patients experienced significant pain, anorexia, persistent vomiting and diarrhea, and profound weight loss which persisted for the entire time from surgery until their deaths. Both spent a significant amount of their remaining days in a hospital or rehabilitation center after surgery. Family members of both of these patients reported doubts over whether surgery was the correct decision for their loved one.

### The Mindset of People Living With Pancreatic Cancer After Surgery

Participants recalled very similar reactions to first receiving their diagnosis of pancreatic cancer. Nearly all participants described intense fear and anxiety when learning about their cancer diagnosis. They noted the stigma that pancreatic cancer carries of being “a death sentence.” This fear was near universal, with no differences noted between participants with different cancer stages or initial treatment approaches (Table 3). Over time, the majority of participants became accustomed to their diagnosis and were able to lead lives without constant fear or anxiety. Only a few participants commented on having persistent feelings of depression or anxiety, feeling as if something was hanging over them, or feeling unable to plan for the future. Anxiety was increased around the time of surveillance imaging, assessment of tumor markers, and doctor’s appointments in general.

**Table 3.** Exemplary Quotes From Patients Demonstrating Their Emotional Response to Receiving a Diagnosis of Pancreatic Adenocarcinoma

**ID**	**Clinical Characteristics**	**Quotation**
02	Stage III Neoadjuvant chemotherapy Distal pancreatectomy	“I was very, very, very scared, because the percentages were so low. Everything was against me having any sort of positive outcome.”
03	Stage IA Neoadjuvant chemotherapy Pancreaticoduodenectomy	“I thought I was doomed. Not many people—and it’s getting better all the time, but I mean, that was the death knell, pancreatic cancer. Everybody I’ve known that ever had it, deceased.”
07	Stage IB Pancreaticoduodenectomy	“I immediately get on the internet and scare myself to death and I was making final preparations. I was, you know, reaching out to family and friends and I fully expected to not survive this. So depression, anxiety, fear, all of those kinds of things kind of swirled around.”
08	Stage IIB Pancreaticoduodenectomy	“I mean, this was basically a death sentence. So, how do you overcome that?”
11	Stage IB Neoadjuvant chemotherapy Pancreaticoduodenectomy	“It wasn’t good. I mean, the first thought that come to my mind is “It’s a death sentence.”
12	Stage IB Pancreaticoduodenectomy	“It was kind of frightening to—when I first got this diagnosis, it was really quite scary, because, you know, you just think, “Well, I’m a goner.”
16	Stage III Neoadjuvant chemotherapy Distal pancreatectomy	“Pancreatic cancer as a whole, statistically, is basically a death sentence. It just depends on how soon or late it’s going to happen.”
17	Stage IIB Neoadjuvant chemotherapy Pancreaticoduodenectomy	“It’s a very emotional time, because—just because of the, you know, the stigma that pancreatic cancer has. With that name, it’s—you hear that, and it’s like okay, here’s your death certificate, you know? Because not very many people survive it.”

Participants’ mindsets varied widely following diagnosis and they reported multiple different responses to living with cancer, including acceptance, denial, depression, and positive thinking (Table 4). Other participants reported approaching their cancer diagnosis as a fight, and used this type of thinking to motivate themselves as they approached surgery, surgical recovery, and adjuvant therapies. For almost all participants, their mindset evolved significantly throughout their time living with cancer and undergoing treatment (Table 5).

**TABLE 4.** Exemplary Quotations Showing Different Responses to Living With Cancer

**Mindset Towards Living with Cancer**	**Quotation**
Depression	“Well, I do have some depression once in a—episodes of it. I’ve come to terms with it. I almost feel like my life as I knew it before is gone. And I’m just living on borrowed time…Any time I plan on something that’s going to take more than a year. I just don’t—I just put it out of my mind, because I don’t feel like I’m going to be around that much longer…I can’t really plan on a future.”—Participant 13
Acceptance	“My sister died when she was [young], and I think very early on I realized that life was not extensive, it was finite, and so…the fact that I had the diagnosis that I have, it’s okay, it happens. So I didn’t freak out, and I still am not freaking out, and I know that when it comes to—life comes to an end. So as far as changing my life or life experience or outlook on life, I don’t think it has. It’s not something I really want, but I’ve got it, so let’s deal with it.”—Participant 01
Denial	“I don’t think I really want to know. I mean, as long as things keep coming back—I mean, you see things that says “If you’ve had this cancer you should read about this.” I don’t read about that. I’m dealing with it, and I don’t want to know all this other stuff, because I think then it gets in your head, and then you start worrying more, and so I just don’t deal with that, and I’m glad the doctors are keeping an eye on things as far as them telling me everything and that it can come back here or there. I don’t particularly want to know that until it happens, if it happens, and then we’ll deal with it at that point.”—Participant 11
Positive Thinking	“When I was going through my therapy, I had a tumor on my pancreas they had to cut out and every day I would think about that and I would think about it as a green blob. Every day, I’d think about that tumor getting smaller and smaller and smaller and tell myself, it’s getting better. Every day, you’re getting better. Yeah, and I think it worked.”—Participant 04
Fighting Attitude	“Well, I’m obviously facing death and so, I’ll do whatever it takes to avoid that and I mean whatever it takes and I’m not going to get tired.”—Participant 08

**TABLE 5.** Exemplary Quotations Demonstrating the Evolution of Individual Participant’s Mindsets As They Progressed Through Their Diagnosis and Treatment

**ID**	**On Diagnosis**	**During Treatment**	**On Recurrence or the Threat of Recurrence**	**On Mortality**
2	“I was very, very, very scared, because the percentages were so low. Everything was against me having any sort of positive outcome.”		“It’s been six months, probably, that [my tumor markers] have been going up, and [my oncologist] just told me that. That would put a hole in your balloon and take some hope away, which it has, but we can’t all live forever. I don’t know. You’re asking me a hard question. Maybe I don’t want to know.”	“I was so thankful. Look at my prognosis and look at my—the ratio of people that can even have surgery. No, I just feel so thankful, and I should’ve been gone a year and a half ago, according to the guys around here. I am so thankful. I am so thankful, and I feel so blessed. I can’t tell you. If it ends tomorrow, that’s still what I would say.”
3	“I just figured I’d—the cancer was going to get me and I might as well accept the fact. All I had hopes was that I could go out swinging. I don’t want to—like I said, I don’t want to lay in bed and just die by inches.”	“He said it would be tough. I didn’t realize quite how tough it was going to be. I don’t know how brave I might have been if I’d have known how miserable I was going to be. Now, I’m glad I did it.”		“I’ve got a month and a week or two and I’ve got a year out of it. So, I can’t bellyache too much and I’ve been functionable for the year. That’s the good part. If I was laid up in bed miserable for a year, I wouldn’t care if I’d made it or not. Right now, I don’t feel bad about it. My family certainly appreciates my being around.”
4	“Well, it was kind of shocking. I’d been a very healthy person my whole life, but then I asked myself, why not me? There’s nothing special about me that I wouldn’t get cancer or a cold or stub my toe…I went through a phase where, poor, poor, pitiful me. Why is it happening to me? It didn’t last very long. Maybe when I first started chemo, it was so altering about what your body is doing to you and like, my gosh.”	“In my mind’s eye, I don’t have any cancer at all… There’s two things, and I call it my PP, the power of prayer and positive thinking. That’s what I feel will help me through this crisis that I have. I’m a better person for it.”	“We talked about [recurrence] last time I met [my surgeon]. It was my one-year anniversary of being cancer free. I said, I made it, and he looked at me like, well, you made it a year. He said, but if you can make it through five years, you’ll be scot-free. You won’t have to worry about these cancers coming back or anything so he painted a good picture but it wasn’t one I was hoping for. If I can make it here, I’ve got it. So anyway, yeah, I’m always thinking about my situation being better than what it is and him going, yeah, you’re not out of the woods yet. You’re still walking in the woods, so it kind of put me down to my feet touching the ground like, come on, kid. You’re not through this yet…I mean, I’m a fighter. I don’t give up on things. I try to see them through to the end, and if I don’t make it, well, then I’ll be part of the 85 percent or 80 percent that didn’t, but so far, I’m doing good. I’m having a good life.”	“I’ve always been optimistic. I’ve always looked at things in respect of, if I can’t change this situation, then how can I change my attitude about the situation, like with the operations and being fatigued and all that crap. It’s just that I’ve had to change my mindset even more because I know that with this type of therapy that I had, it can consume you… I won’t let myself go into that depressed state. I can feel myself sometimes wandering there, but I simply tell myself that’s not what I want. I don’t want my whole life to be determined by what’s happening to me. I just think about, okay, what’s going to happen next to me, in a very positive way…During part of that chemo, I let myself get down on it because I wasn’t feeling worth crap for months and months and I said, now, I’ve got to get myself out of this hole because a person can die happy, too. You don’t have to always be sorrowful, so that’s what’s going to happen. When I die, it’s okay.”
11	“I think I thought of Patrick Swayze at the time because that’s what he had, and so it was pretty tough, and then you have to accept it. There’s nothing you can do to change it, and so you can either feel bad all the time or you just figure “Well, you got to do what you got to do,” and that’s kind of what I did. It knocked me down pretty hard there for a while…, but it’s one of them things that you just have to go on with.”	“I think good. I mean, you got to deal with what you’re dealt with, and for the most part I think I’ve done okay with it. It doesn’t mean that it’s never off your mind, because you think about it, and I go for the scans every six months, and you’re hoping that when you go back that they don’t find anything, but for the most part I just let it go and try to go on.”	“I don’t think I really want to know. I mean, as long as things keep coming back—I mean, you see things that says “If you’ve had this cancer you should read about this.” I don’t read about that. I’m dealing with it, and I don’t want to know all this other stuff, because I think then it gets in your head, and then you start worrying more, and so I just don’t deal with that, and I’m glad the doctors are keeping an eye on things as far as them telling me everything and that it can come back here or there. I don’t particularly want to know that until it happens, if it happens, and then we’ll deal with it at that point.”	
13	“I had an older brother that died of pancreatic cancer three years earlier. And so, I kind of expected a little more drastic thing than what I went through. His was discovered quite later on, and so he didn’t have much chance.”	“One of the main questions that I asked the doctors is, “What kind of a quality of life was I going to have?” If the surgery was just going to extend my life without having any kind of quality to it, I would have said, “No.” But they gave me assurance that I could have—I might not be able to do everything that I wanted, or that I had been doing in my life, but I could still have a quality-of-life, and I’m still looking for that.”		“Well, I do have some depression once in a—episodes of it…I still feel like in many ways it has changed my life completely. And I just—I feel like that I don’t have a future and I have a hard time dealing with that.”

While participants had different coping mechanisms for living with pancreatic cancer, they were unified in the belief that the choice to undergo surgery was the correct decision. Participants, regardless of outcome, had no regrets about the decision to undergo surgery. Even the patients with significant, life-limiting symptoms or early recurrence were satisfied with their decision to receive surgery (Table 6). Family members of the patients who died from their disease or complications of their surgery reported that their loved ones did not regret undergoing surgery. Quantitative data was congruent with qualitative findings. Of 11 participants who completed the DRS, 10 had a DRS score of 0 (no decision-regret), and 1 had a score of 10, demonstrating a high degree of decision-satisfaction.

**TABLE 6.** Exemplary Quotes From Patients Demonstrating Both their Lifestyle-limiting Symptoms and Their Satisfaction With the Decision to Pursue Surgery

**ID**	**Patient Details**	**Lifestyle-limiting Symptoms**	**Satisfaction With Surgical Outcome**	**DRS Score**
03	Surgery: PD Interview at 11.8 mo Recurrence at 4.9 mo Alive	“For two months, I didn’t eat, drink, nothing, laid around in my recliner and bellyached, but I wasn’t hungry and I wasn’t thirsty” “I do stiff physical work, but I don’t last only an hour or two. I’m short of breath. So, it’s an ongoing thing, but I have no stamina whatsoever.”	“I’m limited but it beats the hell out of being in the ground.”	0
13	Surgery: PD Interview at 16.6 mo No recurrence Alive	“Well, to tell you the truth, I felt like my life had ended with the diagnosis. The things that I normally did, I can no longer do…as far as my daily routine and everything, it pretty much stopped.”	“But my biggest concern was, ‘Am I going to be able to just survive? Or am I going to actually still enjoy life?’ And I have. I’ve learned to—it’s changed my attitude in a lot of things.”	10
14	Surgery: PD Interview at 10.3 mo No recurrence Alive	“The way that I’m limited since the Whipple procedure has been because of the digestion issues that I still have…so I stick a little closer to home.”	“Well, number one, I’m lucky that I’m still here.”	0
15	Surgery: PD Interview at 12.3 mo with patient spouse No recurrence Died at 1.2 mo	“I think it was just downhill a little bit every day.”	“I think he would’ve said, ‘Yeah, I want to do it.’ Yeah. Yeah. He wanted to take that chance. That he knew how big that surgery was and he knew the risks and everything and he was willing to do it.”	N/A
17	Surgery: PD Interview at 6.7 mo No recurrence Alive	“I just—you know, I keep an extra pair of underwear, you know, in the truck with me, and, you know, things like that, because sometimes it just—but I am a lot more cautious of how far I get away from, you know, from the toilet.”	“I’ve come to the conclusion that my new normal is what I’m dealing with. I’m not going to get back to—you know, back to where I was, pre-surgery, as far as my digestion and gas and loose stool and all this and that. That’s just something I think is going to be something I’m going to be dealing with for the rest of my life. But I’m still here, so, okay.”	N/A

The final column lists patients’ total score on the decision-regret scale survey.

mo, months; PD, pancreaticoduodenectomy.

Participants reported their high degree of satisfaction came from the opportunity to continue their lives. While a couple participants mentioned they wanted to be alive to see a grandchild get married or graduate from college, most participants were not focused on living to see a certain event or check items off of a bucket list. Participants described everyday life as precious and valued any extra time as valuable. Descriptions of participants typical days focused on finding enjoyment in mundane activities, “a nice cup of coffee,” “just laying back,” “housework or sewing or cooking or whatever,” and “just my regular routine.”

The opportunity to have more time to spend with friends and family was also mentioned frequently as a reason for high decision-satisfaction. Nearly all participants reported that their relationships with family and friends were strengthened following their diagnosis of pancreatic cancer. For some, this came from increased physical and emotional support during medical and surgical treatment. For most however, this increased appreciation came from a change in patient mindset, values, and priorities. Participant 02 stated, “It hasn’t affected anything except me being more aware of my great friendships and loves and families. You don’t get to say that stuff so often…, it’s just better for that.”

### Perceptions on Medical Care and Communication

Participants reported different levels of satisfaction with the information they received preoperatively. Participants were generally satisfied with the amount of information they received on the topic of overall prognosis, including conversations on goals of care and the risk of recurrence or death from disease. Participants also remembered a large portion of their conversations with their surgeons being technical, describing the surgery itself. The one area where participants routinely wished they had more information was the details of what recovery would look like—not the hospital stay and first 2 weeks, but what life would look like 6 months postoperatively. Participants wanted more information on how their lives would change following pancreatic resection. They wanted to know that their digestion, bowel movements, and energy levels would likely not return to normal after surgery.

More detailed information on life after pancreatic surgery would not have changed the decision to proceed with surgery for participants. They did report that they would have felt more prepared for what was to come however. Regarding decision-making, most participants felt like they did not have much control over the decision to proceed with surgical resection. They reported that their surgeons tried to involve them in decision-making and allowed them to have the final say in what treatment course they pursued, but most felt like there wasn’t truly a decision to make. Either they felt like the decision to proceed with surgery was a “no-brainer,” as the alternative was certain death, or they were so overwhelmed by their diagnosis and all of the information they received that they simply followed orders from their doctors about what to do. Overall, despite the caveats mentioned above, participants were overwhelmingly very satisfied with the conversations they had with their surgeons in the preoperative period.

There was little correlation between participants’ responses to the CPS and SDM questions and their interview responses (Table 7). Responses to both surveys fell within a narrow range. SDM mean responses per item ranged from 3.9 to 5.0. CPS responses were all either B, C, or D. These responses did not consistently match with participant comments.

**Table 7.** Comparison of patient responses to interview questions on decision-making and the SDM and CPS survey questions.

**ID**	**Quotation**	**SDM Score**	**CPS Choice**
2	“I was just getting in a line and, “Do this, this, this,” and I can’t remember a whole lot of it, because it was all so new, and I just did it. I just did it, because somebody told me to.”	4	B
4	“Well, I think I had two offers. I could do nothing about it and end up dead, or I could have faith in what they was going to do… Let’s get ‘er done.”	4	D
7	“I didn’t think it was going to be that drastic of a life change. So I was prepared but really wasn’t. I didn’t understand all the ramifications of it…I wish I’d have understood more about it. I did finally grasp the meaning of, okay, this is going to alter your life forever, when I come to that realization.”	4.3	C
8	“Well, I’m obviously facing death and so, I’ll do whatever it takes to avoid that and I mean whatever it takes and I’m not going to get tired.”	4.8	C
13	“I just felt like a lamb being herded from one place to the next. And so, I didn’t open my mouth. I didn’t know what to expect to tell you the truth. And although they told me what the surgery was like, and what they were going to do, I didn’t realize the severity of the—the complexity of the surgery until afterwards.”	5	B

## DISCUSSION

In this study, we examined patients’ lived experiences following pancreatic resection for PDAC with attention to their QOL, mindset, and decision-satisfaction. We identified 4 major themes. First, most patients experience persistent, lifestyle-limiting symptoms following pancreatic resection; however, patients adapt to these symptoms and are content with their “new normal.” Second, the mindset of patients living with PDAC varies widely and evolve significantly as patients proceed through treatment. Third, patients are overwhelmingly satisfied with the decision to pursue surgery. The chance to prolong life is worth significant risks. Fourth, patients with PDAC eligible for surgery most often want the treatment that will offer the greatest chance at cure and are content to trust their medical providers to make treatment decisions for them. These data provide a more nuanced understanding of patient experiences following pancreatic resection than prior literature. The results should be validating to surgeons who perform these operations. Despite the complexities of decision-making for these patients and the overall poor outcomes associated with PDAC, no patient in this cohort reported regret over their decision to pursue surgery.

This cohort of patients described QOL differently than reported by prior quantitative studies. Prior research evaluating QOL in pancreatectomy patients showed that patients see a decline in their wellbeing immediately after surgery with a return to normal by 6 months postoperatively.^16–18^ This cohort of patients did not describe a return to baseline health 6 months postoperatively. Rather, participants described learning to live with persistent symptoms, mainly fatigue and digestive issues, and adapting to a new normal. Importantly, these persistent symptoms and adaptations did not lead to decision-regret for patients. This is consistent with other studies that demonstrated very low rates of decision-regret following pancreatic surgery for PDAC.^19,20^ In this study, understanding the mindset of patients helped to interpret the impact of these persistent symptoms on patients’ overall lived experiences. Participants in this study described how they were willing to live with the consequences of surgery because of the benefits (or chance for benefits) they received. Persistent diarrhea or fatigue are an acceptable price to pay for the chance to prolong life. When facing a lethal diagnosis, patients are willing to adapt to a decreased QOL in order to continue experiencing the small joys of life, their everyday activities, and long-standing relationships.

These findings can help surgeons tailor preoperative conversations with patients to better prepare them for living with PDAC following pancreatic resection. Overall, patients were satisfied with their involvement in decision-making and the information they received about surgery preoperatively. However, patients were not focused (preoperatively or postoperatively) on the technical aspects of surgery, their overall prognosis, or their risks of recurrence or death. Participants were more concerned with understanding how their lives were likely to change following surgery. When discussing surgery with future patients, surgeons may benefit from less focus on the technical aspects of surgery and a greater emphasis on the long-term consequences to QOL of pancreatic resection. Explaining that persistent changes in gastrointestinal function and energy levels occur for many patients may help these future patients better prepare for surgical recovery and their lives following surgery.

Additionally, this study demonstrates how patients feel about decision-making in this setting. Even when they felt disconnected from the decision-making process, participants were very satisfied with their care, happy to trust the judgement of their surgeons. Patients facing a decision about whether to undergo surgery for malignancy want to be participants in the decision-making process; however, they are often overwhelmed by the large amount of information they need to understand.^21^ Many patients do not want to participate in shared-decision making in this context. Rather, they prefer being guided by their surgeons to whatever treatment option provides the best chance for cure. Several other qualitative studies have also questioned the need for shared-decision making in this context.^22–25^

This study must be interpreted in the context of several limitations. First, all participants were recruited from a single-center with 2 highly experienced pancreatic surgeons. Outcomes from other centers or surgeons with different communication styles may differ. Additionally, participants who did not speak English were excluded, further limiting the generalizability of our study findings. Second, this study does not include perspectives of patients who were eligible for surgery but chose not to proceed. Future study should include this cohort to understand differences in how these patients think about the risks and benefits of pancreatic surgery for a cancer diagnosis. Third, all interviews occurred at least 6 months following surgery, and this creates a possibility for recall bias. Fourth, detailed questions on neoadjuvant and adjuvant therapies were not asked in detail, and the impacts of these other therapies were not fully assessed. In reality, surgical and nonsurgical oncologic therapies are not experienced in isolation and future studies should explore this in greater detail. Finally, patients with more negative experiences or those who regretted the decision to proceed with surgery may have declined to participate. We interviewed 62.1% of all patients who underwent pancreatic resection however, and only 4 patients declined to be interviewed. We are unable to know the experiences of these patients, but we do know that there were no differences between these patients and the included cohort in terms of their clinical or oncologic course.

In conclusion, PDAC is a highly lethal cancer and pancreatic resection, the only treatment that offers the potential for cure, has high rates of postoperative complications and significant impacts on patient QOL. This study helps elucidate how patients experience life following pancreatic resection and allows for a more nuanced interpretation of previously published quantitative data. Patients with PDAC are confronted with death and most are willing to accept great risks for a chance to prolong life. Changes in lifestyle and persistent symptoms following surgery are seen as an adequate tradeoff for this opportunity. Surgeons can use this information to instill confidence in their patients and modify their approach to preoperative counseling to better prepare patients for life following pancreatic resection.

## ACKNOWLEDGMENTS

We would like to thank the patients and their family members for sharing their experiences with us. We would also like to thank Dr. Sean J. Mulvihill for his care of many of the patients in this study and career devoted to improving the lives of patients with pancreatic cancer.

## Supplementary Material


