# Supplementary material for: Patient Perceptions of Decision-making and Quality-of-life Following Surgical Resection of Pancreatic Adenocarcinoma: A Mixed-methods Study
Source: Ann Surg Open. 2022 Oct 25;3(4):e214. doi: 10.1097/AS9.0000000000000214 (PMC9780039; doi:10.1097/AS9.0000000000000214)
Supplement: Supplementary file 1 [file as9-3-e214-s001.pdf]

Quality of Life Following Surgical Resection of Pancreatic Adenocarcinoma: a Mixed Methods Study

1. Tell me about your typical day?
    a. How is this different than your typical day before you knew you had cancer?
    b. How did this change after your surgery?
2. How would you describe your overall health?
3. What was your experience of your physical recovery from surgery?
4. How long after surgery did you have pain? Has this changed over time?
5. Did you, or do you still, experience any gastrointestinal symptoms after surgery such as nausea, vomiting, bloating, diarrhea, constipation, or poor appetite? How has this changed over time?
6. Did you experience other concerning physical symptoms after surgery? How have these changed over time?
7. Has your energy level changed following surgery?
8. Have you had issues with memory or concentration since surgery?
9. Has your ability to function on a normal day changed since surgery? Are you still able to do the things you want to do?
10. Would you describe how you were affected emotionally through the whole process? Did you experience any emotional challenges after surgery such as depression, anxiety, or other mood changes? Has this changed over time?
11. Were your relationships with loved ones and friends affected by your surgery?
12. How did surgery effect your work life, family life, leisure activity, social life?

13. What was your response to first learning you had a diagnosis of cancer?
14. Tell me about your experience when you first met with your surgeon?
    a. What were your emotions going into this meeting and how did they change after?
    b. Did you get all of the information you were hoping to out of this meeting? Were you able to retain the information you were given?
    c. Were your concerns about your overall prognosis addressed?
15. Do you think you were prepared for your surgery?
16. Do you think you understood your prognosis before surgery? What was your emotional response to this?
17. When making decisions about treatment, would you say you prefer an active role, passive role, or collaborative role?
    a. Do you feel you had the control you wanted over the decision to undergo surgery?

18. Were your expectations of surgery consistent with what you actually experienced?
19. If you were to go back to that first day you met with your surgeon, would you change anything about this whole process?
20. What advice would you give to someone who was about to undergo the same surgery you had?
21. Knowing what you know now, would you do it again?

22. Is there anything else on this topic you feel is important but we haven’t discussed yet?
